# Full-thickness skin regeneration beneath the exposed titanium mesh in cranioplasty: Two cases report

**DOI:** 10.1097/MD.0000000000034821

**Published:** 2023-08-18

**Authors:** Daohong Kan, Xuefeng He, Bing Liu, Chaokun Yang, Yong Zou

**Affiliations:** a Department of Burn and Plastic Surgery, The Second People’s Hospital of Yibin (The Yibin Hospital of West China Hospital, Sichuan University), Sichuan, China; b Department of Burns and Wound Repair, The First Affiliated Hospital of Sun Yat-Sen University, Guangzhou, China; c Department of Cardio Thoracic Surgery, The Second People’s Hospital of Yibin (The Yibin Hospital of West China Hospital, Sichuan University), Sichuan, China.

**Keywords:** case report, cranioplasty, implant exposure, skin regeneration, titanium mesh

## Abstract

**Rationale:**

Titanium mesh is one of the most widely used implant materials applied in cranioplasty; however, it has been reported to encounter the risk of progressive scalp thinning and implant exposure over time. Here we present 2 cases of exposed titanium mesh (TM) and unusual phenomena of full-thickness skin regeneration beneath the mesh.

**Patient concerns:**

Two patients, 1 with an 8-year and 1 with a 2-year history of implant exposure after cranial TM implantation.

**Diagnoses:**

The patients were diagnosed with scalp ulcers and cranial TM exposure.

**Intervention:**

The exposed part of the implant was removed, and the full-thickness skin beneath the mesh was directly used as functional soft tissue coverage to repair the scalp defect.

**Outcomes:**

Full recovery for both patients with cosmetic satisfaction.

**Lessons:**

Though the exact mechanism of this epithelisation phenomenon beneath the TM remains to be elucidated, it provided a feasible choice for clinicians to reconstruct the scalp’s integrity without exerting complicated procedures when dealing with similar cases.

## 1. Introduction

Cranial defect secondary to congenital deformity, traumatic injury, osteoradionecrosis, oncological ablative procedure or decompressive craniectomy usually needs a second-stage cranioplasty to reconstruct the normal neurocranial anatomical boundary.^[1]^ So far, plenty of alloplastic materials are being applied in cranioplasty; in general, titanium mesh (TM) is prioritized over other materials for being light, rigid but easily manipulatable, biologically inert, and low cost.^[2–4]^ However, recent studies emphasized that progressive skin thinning above the mesh was frequently discovered in many patients who received cranial TM implantation, and the subsequent implant exposure seemed inevitable over time.^[1,5–7]^ Nevertheless, the underlying mechanisms remain to be elucidated.

Here we present 2 cases of full-thickness skin regeneration beneath the TM after long-duration implant exposure. A similar case was reported by Steven Liben Zhang in 2021, and the skin regeneration process has been briefly described as “dermointegration,” demonstrating a possible protective phenomenon after cranioplasty using TM as reconstructive material.^[8]^

## 2. Case report

### 2.1. Case 1

**Figure 1.**
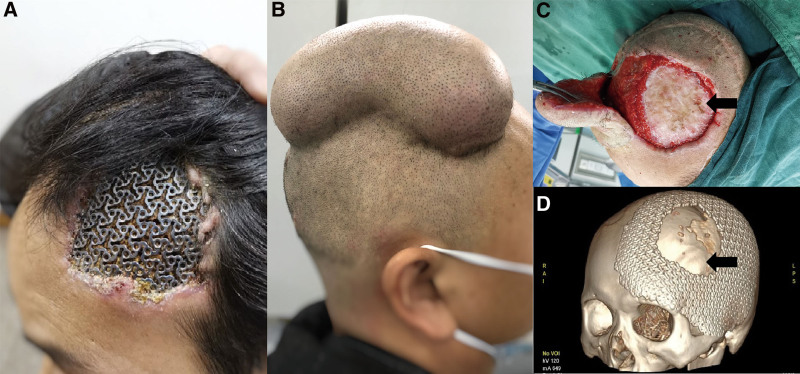
(A) Physical examination at admission revealed a frontotemporal scalp defect and titanium mesh exposure (12 cm × 10 cm). (B) A kidney-shaped tissue expander was implanted under the vertex scalp at the first stage of reconstruction surgery. (C) Removing the exposed part of the implant revealed a continuous epithelial layer (full-thickness skin with hairs) underneath (black arrow). (D) Postoperative 3D-CT demonstrated the removed part of the implant (black arrow).

A 38-year-old male patient presented with cranial TM exposure had been admitted to our department. Eight years before admission, the patient had undergone cranioplasty after a traumatic decompressive front parietotemporal craniectomy, and implant exposure occurred shortly after the surgery. The patient lived with exposed mesh for 8 years without seeking any medical treatment and did not experience any discomfort or accompanying symptoms. Physical examination at admission revealed a 12 cm × 10 cm scalp defect and exposed implant in the left frontotemporal area (Fig. 1A).

A 2-stage reconstruction plan was determined based on the patient’s conditions.^[9,10]^ During the first stage of soft tissue expansion, a kidney-shaped tissue expander was implanted under the vertex scalp (Fig. 1B); the second-stage reconstructive operation was performed 6 months after the implantation. Restricted by the vast gap between the implant and the dura mater, it was challenging to perform strict debridement through the venting holes of the mesh. We first had to remove the exposed implant to avoid secondary infection and reconstruct the scalp’s integrity. Surprisingly, an unusual phenomenon was observed beneath the implant, and the dura mater appeared covered by full-thickness skin with appendages (Fig. 1C).

**Figure 2.**
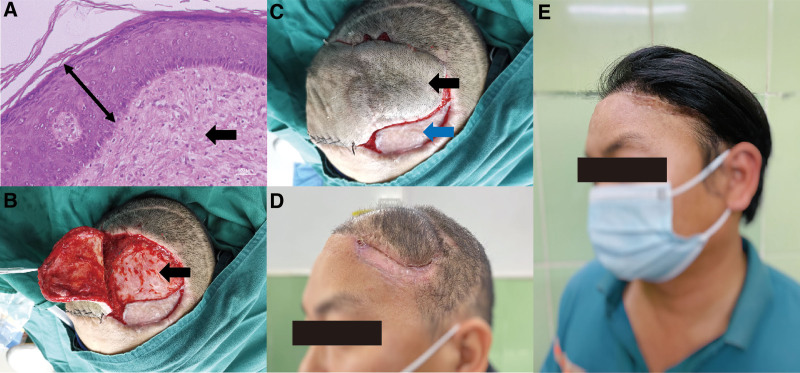
(A) Histopathology results (H&E staining, ×20, bar = 50 μm) demonstrated the full-thickness skin (double arrow) and excessive collagen fibers deposition (black arrow). (B) Part of the covering above the dura mater was removed to provide a wound bed for flap transfer, revealing a rigid connective tissue stratum (black arrow). (C) The defect was reconstructed with the pre-expanded flap (black arrow) and the remaining part of the dura mater covering (blue arrow). (D) The patient was discharged with full recovery after a three-week hospitalization. (E) In a one-year follow-up, the patient showed no morbidities and was satisfied with the cosmetic outcome.

Postoperative 3 dimensional-CT demonstrated the removed part of the implant (Fig. 1D). Postoperative histopathology results confirmed that the outermost covering was full-thickness skin; moreover, muddled collagen fibers formed a rigid connective tissue stratum, bearing a similar structure as hypertrophic scar tissue (Fig. 2A). During the reconstruction delay, the pre-expanded flap began to shrink in size, making it insufficient to cover the whole frontotemporal scalp defect. Hence, part of the covering was removed, exposing subcutaneous tissues, which served as a wound bed for flap transfer, and the remaining part of the covering was directly used as functional soft tissue coverage (Fig. 2B and C). The patient was discharged with full recovery after a 3-week hospitalization (Fig. 2D). In a 1-year follow-up, the patient showed no morbidities and was satisfied with the cosmetic outcome (Fig. 2E).

### 2.2. Case 2

**Figure 3.**
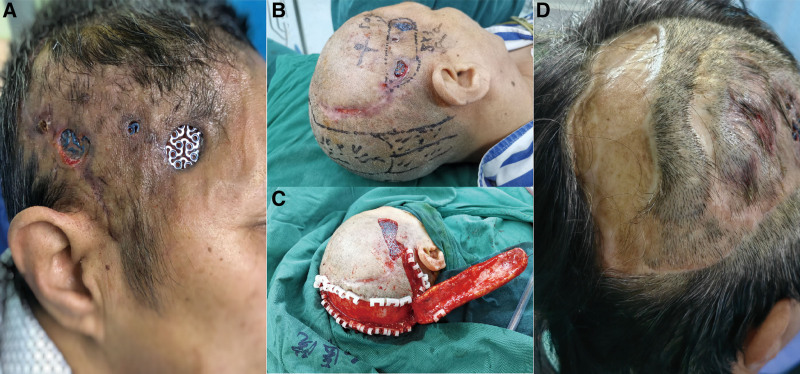
(A) Physical examination at admission revealed some scalp defects and titanium mesh exposure. (B and C) An axial skin flap (occipital artery nourished) was used to repair the scalp defect. (D) In a one-year follow-up, the patient fully recovered from the reconstructive surgery.

**Figure 4.**
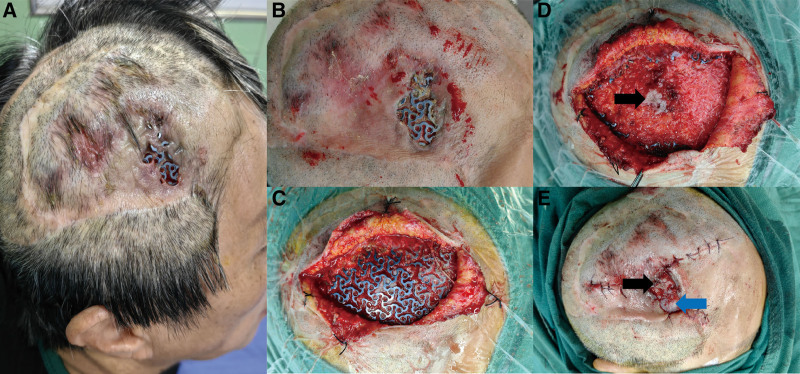
(A) A 3.5 cm × 2.5 cm scalp defect and implant exposure were found in another area one-year after the reconstructive surgery. (B and C) A one-stage plan of removing the exposed part of the implant and repairing the wound with skin grafting was determined. (D) Full-thickness skin was discovered beneath the mesh (black arrow). (E) The skin had been maintained as functional soft tissue coverage (black arrow) supplemented by autologous skin grafting (blue arrow).

A 56-year-old female patient with right temporal TM exposure was admitted to our department. She had undergone cranial TM implantation after a traumatic decompressive craniectomy 2 years ago but presented with partial implant exposure 6 months later (Fig. 3A). In the first repair surgery, the implant had been preserved. An axial skin flap (occipital artery nourished) was used to repair the scalp defect (Fig. 3B and C). One-year later, the patient was fully recovered from the last surgery, yet presented implant exposure in another area with a 3.5 cm × 2.5 cm scalp defect (Figs. 3D and 4A). Considering the systemic condition of the patient and the local condition around the exposed area (thinned scalp), a 1-stage plan of removing the exposed part of the implant and repairing the wound with skin grafting was determined (Fig. 4B and C). Again, full-thickness skin was discovered beneath the mesh, bearing similar characteristics as the case above (Fig. 4D). Based on previous experience, the skin had been maintained as functional soft tissue coverage supplemented by autologous skin grafting (Fig. 4E). The patient was discharged with a full recovery 2 weeks later.

## 3. Discussion

TM is one of the most widely used implant materials for cranioplasty. Recent studies noted that patients with cranial TM were at high-risk of progressive scalp thinning, and accordingly, implant exposure was inevitable over time.^[1]^ The underlying mechanism of the scalp and subcutaneous tissue thinning after TM implantation has not been fully illuminated; nonetheless, some notable hypotheses are being distinguished, including metal hypersensitivities,^[4]^ mechanical friction and the fluctuating pressure gradient between the atmosphere and the intracranial space.^[11]^ Specifically, Nobutaka Yoshioka and his colleagues have proposed a 5-staged hypothesis about the progressive changes around the mesh after cranioplasty based on pressure gradient alteration, which interpreted the primary initiation and progression of the scalp thinning after TM implantation.^[11]^ However, full-thickness skin regeneration was happening beneath the implant simultaneously, and little is known about this phenomenon’s prerequisites and initiation factors. As is well known, the dura mater is the outermost layer of the 3 membranes covering the brain and spinal cord, composed of meningeal fibroblasts.^[12]^ So far, there is no evidence indicating its capacity to regenerate, proliferate or differentiate. Similar cases of long-duration exposed mesh were reported in 2013, and only granulation tissue and new ossification were discovered around the defect when the exposed mesh was removed.^[13]^ Thus, ossification and regeneration of the peripheral skull may contribute to subsequent change under the mesh;^[14]^ yet, it seems insufficient to generate full coverage of the dura mater for medium to large-sized cranial defects.^[15]^

**Figure 5.**
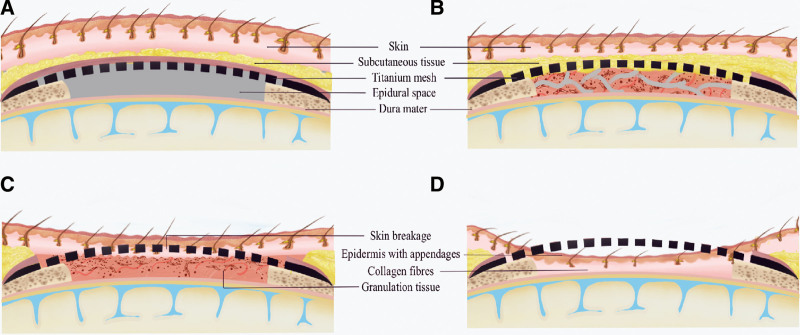
Schematic demonstration of the dynamic and sequential occurrence of scalp thinning, implant exposure, and skin regeneration after cranial titanium mesh implantation. (A) The titanium mesh is primarily placed above the skull, forming an epidural space between the subcutaneous tissue and dura mater. (B) The subcutaneous tissues intrude into the mesh holes, experiencing degeneration, necrosis, organization and resorption, and form a granulation tissue stratum under the mesh. (C) The skin was broken down around the mesh holes; viable cells from pilosebaceous units, eccrine sweat glands and the outer root sheath of the hair follicle landed on the granulation tissue under through the holes. (D) Skin cells coalesced over the underlying dura mater, repopulating, and forming a continuous epithelial layer – a protective barrier between the brain and the external environment.

Based on previous literature, we propose another hypothesis about the dynamic and sequential occurrence of scalp thinning, implant exposure, and skin regeneration after cranial TM implantation (Fig. 5).^[11]^ Usually, the TM was placed upon the skull and fixed with screws in a typical cranioplasty procedure. When the bone defect was large enough to form a vast epidural space under the mesh, the pressure gradient between the atmosphere and the intracranial space initiated subcutaneous tissues to intrude into the mesh holes (Fig. 5A). Along with the progression, the skin and subcutaneous fat grow thinner and thinner in a macroscopic view, causing impending implant exposure. In the meantime, subcutaneous tissues were inclined to experience degeneration, necrosis, organization, and resorption, eventually forming a granulation tissue stratum under the mesh (Fig. 5B). Subsequently, the skin was broken down around the mesh holes; viable cells from pilosebaceous units, eccrine sweat glands and the outer root sheath of the hair follicle landed on the granulation tissue under through the holes (Fig. 5C).^[16]^ Skin cells coalesced over the underlying dura mater, repopulating and forming a continuous epithelial layer – a protective barrier between the brain and the external environment (Fig. 5D).

Though the mechanism is not fully understood for now, some facts need to be noted. Firstly, dermo integration has never been reported before in cranioplasty using other alloplastic material, and it seems to be a customized byproduct only seen in TM implantation.^[5,17]^ Additionally, based on current knowledge, the thinning process is believed to involve the surgical site and defect size. For instance, implant exposures were more frequently found in frontal and temporal defects shortly after cranioplasty, and it could be ascribed to the primarily thinner skin and subcutaneous tissues in these areas.^[5,18]^ As for the defect size, the controversy lies in the fact that TM prioritizes over other alloplastic materials in many aspects, especially for reconstructing medium to large-sized cranial defects; However, a larger size is believed to be negatively correlated with the retention rate of the implant in the long run.^[3,6]^ Last but not least, the configuration of the mesh and the number of mesh holes could also impact the progression of scalp thinning and decide the outcome of cranioplasty using TM.^[11,19]^ Removing only the exposed part of the mesh avoids accompanying morbidities of complex reconstruction procedures, especially for those patients with advanced age or severe comorbidities. In future practice, clinicians should discern high-risk conditions like defect area (the frontal and temporal area) and defect size before performing TM implantation. Thus, better choices can be made to avoid postoperative complications and bring the optimum clinical outcome.^[20]^

## 4. Conclusion

TM exposure is an unexpected and unpleasant complication secondary to cranioplasty; however, in the present cases, the manifestation of full-thickness skin beneath the TM served as a protective barrier against possible damage secondary to implant exposure. Based on previous research, we have proposed a hypothesis about the dynamic and sequential occurrence of scalp thinning, implant exposure and skin regeneration after cranial TM implantation. Accordingly, removing the exposed part of the implant (supplemented with skin grafting) can be a suboptimal but feasible choice when managing patients who suffer from TM exposure but are poor candidates for complicated reconstruction surgery.

## Author contributions

**Conceptualization:** Daohong Kan, Bing Liu.

**Investigation:** Daohong Kan, Xuefeng He.

**Supervision:** Bing Liu, Chaokun Yang, Yong Zou.

**Validation:** Chaokun Yang.

**Writing – original draft:** Daohong Kan.

**Writing – review & editing:** Xuefeng He, Yong Zou.
